# The Evolution of Complex Carbide Precipitates in a Low Alloy Cr–Mo–V Steel after Long-Term Aging Treatment

**DOI:** 10.3390/ma12101724

**Published:** 2019-05-27

**Authors:** Zili Liu, Chunming Liu, Lede Miao, Xiaofei Guo, Jianhua Ding, Hanqian Zhang

**Affiliations:** 1 School of Materials Science and Engineering, Northeastern University, Shenyang 110819, China; 2 Central Research Institute (R&D Center), Baoshan Iron & Steel Co. Ltd., Shanghai 201900, China; miaolede@baosteel.com (L.M.); jhding@baosteel.com (J.D.); hq_zhang@baosteel.com (H.Z.); 3 Steel Institute, RWTH Aachen University, 52072 Aachen, Germany; xiaofei.guo@iehk.rwth-aachen.de

**Keywords:** Cr–Mo–V steel, long-term aging, carbide evolution characterization, planar mismatch

## Abstract

Complex carbide precipitates in a quenched and tempered low alloy Cr–Mo–V steel after long-term aging at 650 °C for 13,000 h and 30,000 h were investigated in this study. The mass fraction and sizes of precipitates were quantified by electrolytical extraction technique. The types of precipitate were further studied by combined X-ray diffraction and transmission electron microscopy with selected area electron diffraction and energy dispersive spectrometry. A series of carbide precipitates, namely MC, M_7_C_3_, M_6_C, and M_2_C, were found existing in the near-equilibrium state. The precipitate sequence of these carbides was identified as MC + M_7_C_3_ + M_2_C → MC + M_2_C + M_7_C_3_ + M_6_C → MC + M_7_C_3_ + M_6_C. It was clarified that the stable phases for the investigated steel aged at 650 °C were composed of MC, M_7_C_3_, and M_6_C. For the first time, the in-situ transformations of M_2_C to M_6_C and M_7_C_3_ to M_6_C were directly observed. It was also observed that the nucleation site of the M_6_C was located at the interface of M_7_C_3_ carbides and the matrix. The orientation relationships between the secondary phases of the in-situ transforming carbides aged for 13,000 h and 30,000 h at 650 °C were established. The coherent interfaces between these secondary phases became incoherent with prolonged aging treatment due to the exerted strain field of the growing carbides.

## 1. Introduction

Low alloy Cr–Mo–V and Cr–Mo steels are creep resistant steels [1], showing excellent mechanical strength at high temperatures. They are extensively utilized in manufacturing key equipment in the power generation industry [2,3] and petroleum industry [4]. Different types of carbide precipitate manifested by aging treatment strengthen these low alloy steels; however, they also influence the microstructure stability [5] and high temperature creep resistant properties [6,7,8,9]. The characterization of carbide precipitates and their contribution to mechanical properties under different aging conditions have been intensively investigated [10,11,12,13,14,15,16,17,18]. Most of these aging treatments were carried out from several hours to several thousand hours at a temperature range between 300 and 750 °C [19,20,21,22,23,24]. Limited work focuses on long-term heat treatment with an aging time long enough to be regarded as approaching the equilibrium state [22]. However, the equilibrium states of the carbides are very important for understanding the degradation or the failure mechanism of the steel, since the material will be continuously exposed to high temperature during service.

Much research work has been carried out to clarify the crystallographic orientation relationship between the precipitates and the ferrite matrix, as summarized in Table 1 [25,26,27,28,29,30]. The crystallographic orientation relationships between the in-situ transformed precipitates and the matrix are crucial for the in-depth understanding of carbide evolution and the mechanisms behind this evolution. Up to now, however, limited investigation has been performed on the orientation relationship between the in-situ transformed carbides in Cr–Mo–V steels [24]. Two nucleation mechanisms for the new carbide phases might work at the stage of aging; one is named ‘in-situ transformation’, which means the new carbide directly nucleates from the existing carbide and grows at the expense of the mother phase; another mechanism is called ‘separate nucleation’, which means the existing carbides are dissolved firstly in the base metal and then the new carbide nucleates and grows. Baker and Nutting postulated the in-situ transformation from M_2_X (M_2_C) to M_6_C according to their indirect observations since 1959 [31]. The letter M represents the metallic component, such as Fe, V, Cr, Mo, Mn, etc.; the letter X represents the non-metallic elements, such as C and N. Beech has endeavored to find in-situ transformation by obtaining the electron diffraction patterns from individual precipitate but never succeeded [32]. Then Beech believed that carbide transformed by a mechanism of separate nucleation rather than an in-situ nucleation mechanism. There are several difficulties to get valuable crystallographic information of the in-situ transformed precipitates: first, sufficient aging treatment period to approach the equilibrium state; second, capturing the in-situ transformed complex carbide precipitates among the tens of thousands of precipitates in the aged specimen; third, the precise index of the complex selected area diffraction patterns from the in-situ transformed carbide precipitates.

In previous studies [15,33], when similar steel was aged at about 650 °C, there was usually a discrepancy between the experimental results and the theoretical calculations for the stable phases. Miyata and Sawaragi [34] used a thermodynamic database (THERMO-CALC) to calculate the equilibrium phases after a 650 °C aging treatment, which were supposed to be M_23_C_6_ + MC. Vyrostkova et al. [22] found different results in their calculations, which were supposed to be M_6_C + M_7_C_3_ + MC. Therefore, the equilibrium phases at the aging temperature of 650 °C for the investigated steel are still an open issue from the scientific point of view, which still need to be clarified by experimental observation.

In our study, the selected area electron diffraction (SAED) patterns were indexed according to the crystallographic information of the typical carbides that may precipitate in the studied steel; these basic data are briefly described in Table 2 [35,36,37,38].

In the present study, quenched and tempered low alloy Cr–Mo–V steel was isothermally aged at 650 °C for the period of 13,000 h and 30,000 h, respectively. The type, precipitate sequence, nucleation mechanism, and crystal structure of the carbides, which precipitated during aging, were studied systematically by means of electrolytically extracted technique, X-ray diffraction, and transmission electron microscopy. In particular, the evidences of an in-situ transformation of M_2_C to M_6_C, M_7_C_3_ to M_6_C were directly observed for the first time. In addition, the orientation relationships between the in-situ transforming carbides were demonstrated by means of transmission electron microscopy (TEM) and the selected area electron diffraction (SAED) patterns and energy dispersive spectrometer (EDS). The interfaces between different phases in the complex precipitates were characterized accordingly. The current study will be very helpful to understand the complex precipitation phenomenon during long-term aging treatment of low alloy Cr–Mo–V steel.

## 2. Experimental Procedure

The Cr–Mo–V low alloy steel was melted in a vacuum furnace (Inductotherm, Rancocas, NJ, USA), cast into 150 kg blocks, hot rolled to plates of 50 mm thickness, austenitized at 950 °C for 4 h, and water quenched. The quenched plates were tempered at 720 °C for 150 min and air-cooled. Then the tempered steel plates were aged in a NCPE-411 type furnace (Nakanihon Ro Kogyo, Nagoya, Aichi-ken, Japan) at 650 °C for 13,000 h and 30,000 h, respectively. The chemical composition of the investigated steel is listed in Table 3.

Part of the base metal and the aged plates were machined to cylinder specimens with a diameter of 10 mm and a length of 70 mm. They were subsequently dissolved in a 5% KCl + 1% citric acid distilled water solution to obtain the precipitates by electrolytic extraction technique. The extracted precipitates were filtered using a filter (Millipore, Darmstadt, Germany) with 0.05 μm pores, thoroughly dried at 70 °C, and weighed by electronic balance (Mettler Toledo, Zurich, Switzerland) with the accuracy of ±1 μg. The size distributions of the extracted precipitates were analyzed by a Marstersizer 2000 particle size analyzer from Malvern Instruments Company (Malvern, UK) with a measurement error of ±3 nm.

The extracted particle powder was analyzed by a Bruker D8 discover X-ray diffractometer (Bruker, Karlsruhe, Germany) with CuK α radiation and graphite monochromators (Bruker, Karlsruhe, Germany). The extracted precipitates were analyzed on a piece of glass plate in the air atmosphere. The start angle, end angle, and step size (2*θ*) were 10°, 80°, and 0.02°, respectively, with a count time of 2.0 s per step.

The specimens prepared for transmission electron microscopy (TEM, JEOL, Akishima, Tokyo, Japan) observation were cut from the aged plate, mechanically ground to 80–100 μm, and punched into disks with 3 mm in diameter. The disks were further electro-polished using a solution of 10% perchloric acid in ethanol. A JEM-2100F scanning transmission electron microscope (JEOL, Akishima, Tokyo, Japan) attached with an Oxford energy-dispersive X-ray spectrometer (EDS, Oxford Instruments, Oxford, UK) was used for microstructure observation and carbide identification. Selected area electron diffraction (SAED) was employed for identifying the crystallographic details of the complex precipitates.

## 3. Results

### 3.1. Mass Fraction of Precipitates in Different Aging Conditions

The mass fraction of the precipitates extracted from specimens was calculated by the following formula:

(1) $$f=\frac{m_{p}}{m_{o}-m_{r}}\times 100\%=\frac{m_{p}}{\Delta m}\times 100\%$$

where *m_p_* is the weight of the extracted precipitates of the aged specimen, *m_o_* is the original weight of the aged specimen before electrolytically extraction, and *m_r_* is the remained weight of the aged specimen after the extraction experiment.

**Figure 1:**
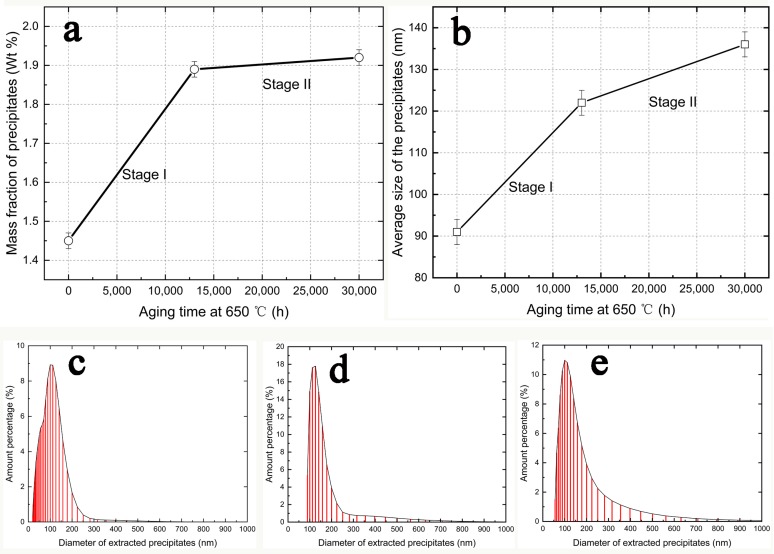
Mass fraction and size distribution of precipitates extracted from the specimens aged at 650 °C for different aging conditions. (**a**) Mass fraction of the precipitates at different aging conditions, (**b**) average size of the precipitates at different aging conditions, (**c**–**e**) size distribution of precipitates extracted from the quenched and tempered specimen, the 650 °C for 13,000 h aged specimen, and the 650 °C for 30,000 h aged specimen.

Figure 1a displays the mass fraction of precipitates in the specimens aged at 650 °C for the applied aging time. The mass fraction of the precipitates increases steeply in the specimens aged at 13,000 h compared to that from the tempered specimen, which was designated as stage І. From the aging time of 13,000 h to 30,000 h, which was defined as stage II, there was only a slight increase in the mass percentage of the precipitates.

### 3.2. Particle Size Distribution

Figure 1b shows that the average sizes of the extracted precipitates were 91 nm, 121 nm, and 136 nm for the 0 h, 13,000 h, and 30,000 h aging treated specimens, respectively. Comparing the average particle size increment of the two stages, the increment of stage II was much smaller than stage І, although the aging time of stage ІІ was longer than stage І. The size distribution of the extracted precipitates was plotted, as shown in Figure 1c–e. Some ultra-fine precipitates may have been lost during the filtering process.

### 3.3. X-ray Diffraction Analysis of the Extracted Precipitates

**Figure 2:**
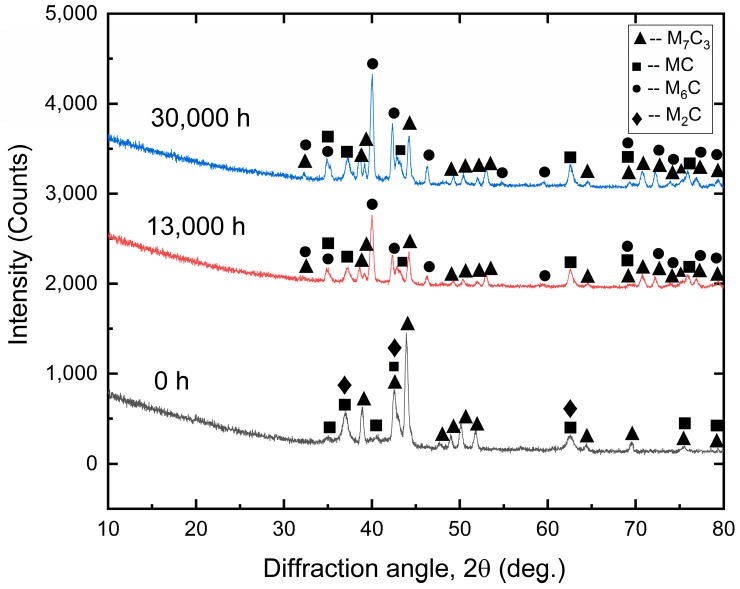
X-ray diffraction patterns of the electrolytic extracted precipitates from different aging conditions.

X-ray diffraction analysis was performed to identify the types and mass fraction of the precipitates from the electrolytic extracted particles. Figure 2 reveals the diffraction patterns of the extracted precipitates corresponding to specimens aged at 650 °C for 0, 13,000, and 30,000 h, respectively. No obvious diffracted peaks were found at the angle below 30 degrees. Table 4 summarizes the detected types of precipitates from the diffraction pattern. The precipitates extracted from the quenched and tempered specimens consisted of M_7_C_3_, M_2_C, and MC. After aging treatment at 650 °C for 13,000 h, the diffraction peaks from M_7_C_3_ became weaker and new diffraction peaks corresponding to the lattice structure of M_6_C appeared. The diffraction peaks from the 30,000 h aged specimens had identical peak angles as the 13,000 h aged specimen. The only difference was that the 30,000 h aged specimen had a stronger peak intensity.

**Figure 3:**
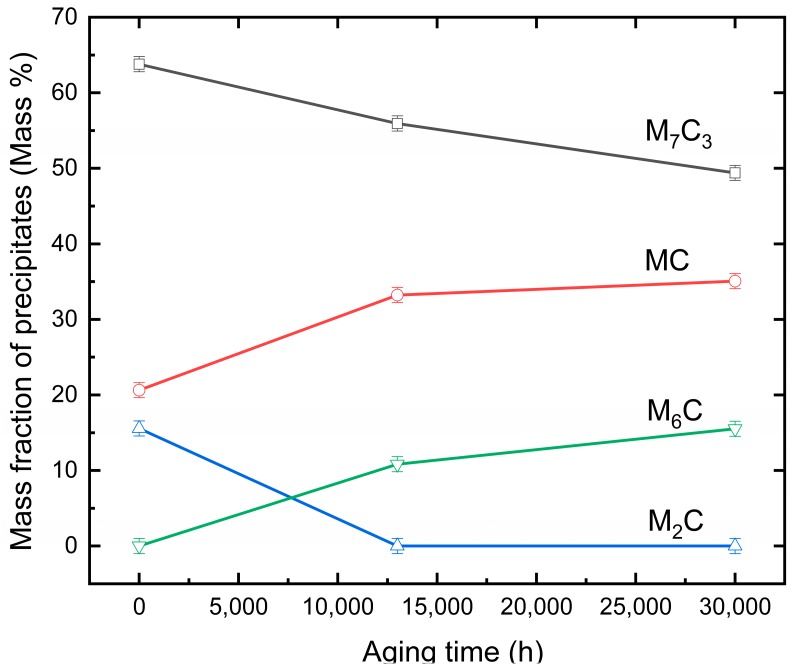
Mass fraction of different types of precipitates with prolonged aging.

The changes of the intensity of the diffraction peaks, to some extent, was a reflection of the change of the mass fraction of each type of precipitate. Figure 3 shows the calculated mass fraction of each type of precipitate by applying the Rietveld full-pattern fitting algorithm method [39]. The TOtal PAttern Solution (TOPAS, version 4.1) developed by Bruker company (Karlsruhe, Germany) was used for the refinement, and weighted pattern R-factor (Rwp) was selected as a criterion to judge the refinement. In the current experiment, the Rwp value was about 8%, which was below the value of 10% for reliable refinement. The amount of M_7_C_3_ type of carbide decreased, whilst the amounts of the MC and M_6_C carbides increased with prolonged aging time. M_2_C disappeared when aging time was longer than 13,000 h.

### 3.4. TEM, SAED, and EDS Characterization of the Crystallographic Orientation Relationship of the In-Situ Transformed Carbides 

**Figure 4:**
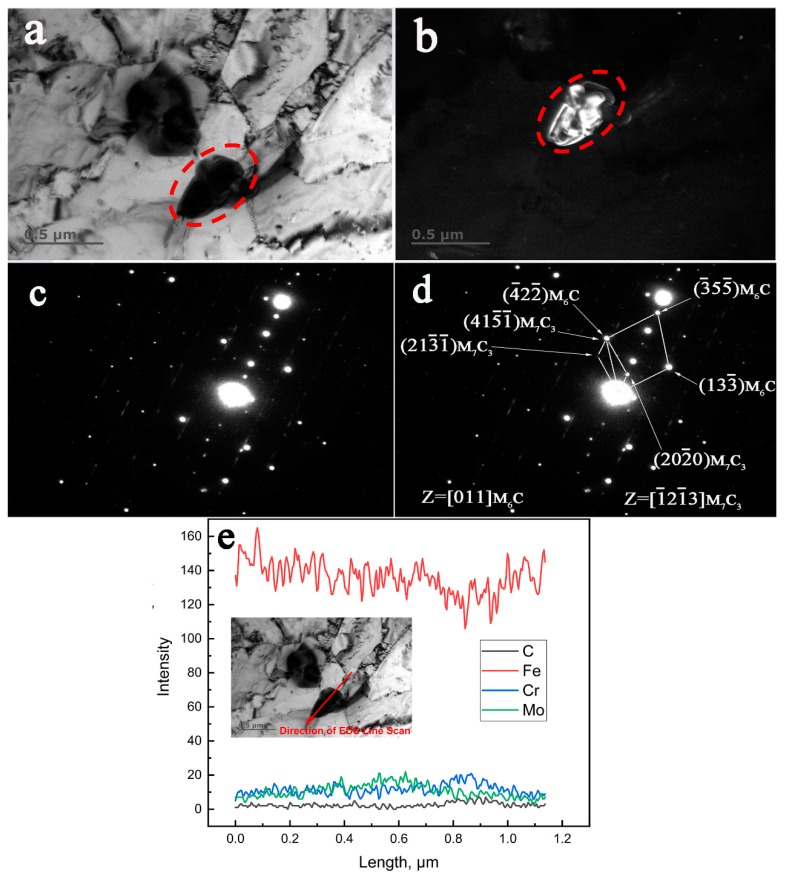
TEM characterization of the in-situ transformation of M_7_C_3_ to M_6_C in the low alloy Cr–Mo–V steel aged at 650 °C for 13,000 h. (**a**) BF image, (**b**) DF image, (**c**) the original selected area electron diffraction (SAED) pattern, (**d**) the corresponding indexed SAED pattern, (**e**) the energy dispersive spectrometer (EDS) line scan.

Figure 4 reveals the transformation from existing M_7_C_3_ to M_6_C in the specimen aged at 650 °C for 13,000 h by combined TEM, SAED, and EDS characterization. Figure 4a,b display the BF (Bright-Field) and DF (Dark-Field) TEM images of the M_7_C_3_ + M_6_C complex precipitate. The spot used for the conventional dark field image was ${\left(\bar{4}2\bar{2}\right)}_{M_{6}C}$. The SAED patterns, as seen in Figure 4c, revealed that the diffracted spots of different phases tangled together in the same image, which was completely different from the ordered pattern of a single phase. Figure 4d shows the indexing of the diffraction spots of the SAED patterns in Figure 4c. It indicates that the transformation of M_7_C_3_ to M_6_C took place according to an in-situ mechanism. The crystallographic orientation relationships between the M_7_C_3_ and M_6_C obtained from the SAED pattern (Figure 4d) were identified as follows:

$$\begin{array}{l} {\left(41\bar{5}\bar{1}\right)}_{M_{7}C_{3}}//{\left(\bar{4}2\bar{2}\right)}_{M_{6}C}\\ {\left[\bar{1}2\bar{1}3\right]}_{M_{7}C_{3}}//{\left[011\right]}_{M_{6}C} \end{array}$$

Figure 4e shows the distribution of the main elements in the complex precipitates along the direction of the arrow in the inserted picture in Figure 4e. It reveals that the precipitate definitely consisted of two different particles; one was rich in Mo (M_6_C) and the other was rich in Cr (M_7_C_3_).

**Figure 5:**
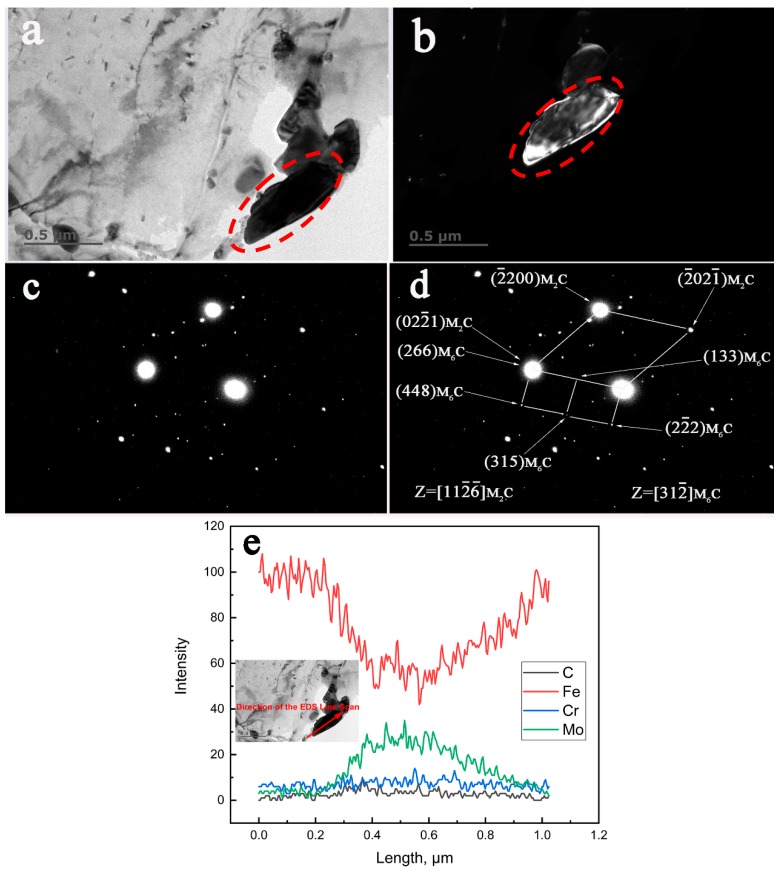
TEM characterization of the in-situ transformation of M_2_C to M_6_C in the low alloy Cr–Mo–V steel aged at 650 °C for 13,000 h. (**a**) BF image, (**b**) DF image, (**c**) the original SAED pattern, (**d**) the corresponding indexed SAED pattern, (**e**) the EDS line scan.

Figure 5 reveals an example of the transformation from existing M_2_C to M_6_C in the specimen aged at 650 °C for 13,000 h by combined TEM, SAED, and EDS characterization. The BF and DF images from Figure 5a,b reveal the comparable size of the precipitates described in Figure 4. The spot used for the dark field image in Figure 5b was ${\left(133\right)}_{M_{6}C}$. The orientation relationships between M_2_C and M_6_C as indexed in Figure 5d are presented as follows:

$$\begin{array}{l} {\left(02\bar{2}1\right)}_{M_{2}C}//{\left(266\right)}_{M_{6}C}\\ {\left[11\bar{2}\bar{6}\right]}_{M_{2}C}//{\left[31\bar{2}\right]}_{M_{6}C} \end{array}$$

Figure 5c is the original SAED pattern of the studied precipitate. As shown in Figure 5e, it is clear that this carbide was rich in Mo and poor in Cr.

**Figure 6:**
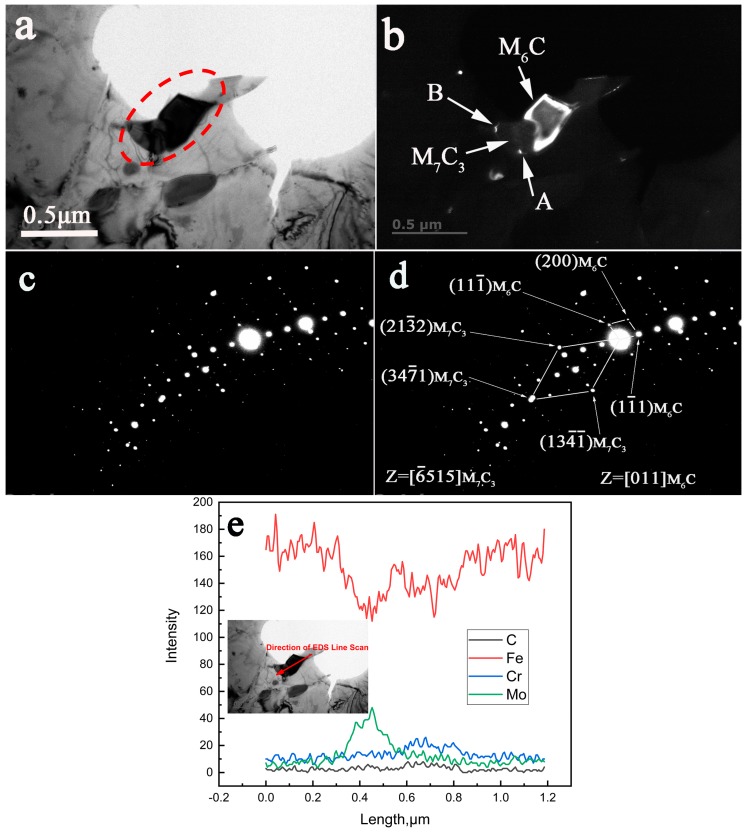
TEM characterization of the in-situ transformation of M_7_C_3_ to M_6_C in the low alloy Cr–Mo–V steel aged at 650 °C for 30,000 h. (**a**) BF image, (**b**) DF image, A and B represent the small white precipitates near M_7_C_3_, (**c**) the original SAED pattern, (**d**) the corresponding indexed SAED pattern, (**e**) the EDS line scan.

The in-situ transformation from M_7_C_3_ to M_6_C and from M_2_C to M_6_C was also observed in the specimen aged at 650 °C for 30,000 h. The spot used for Figure 6b was ${\left(1\bar{1}1\right)}_{M_{6}C}$. As seen Figure 6d, the reflection spots that were diffracted from M_7_C_3_ and M_6_C could be seen in the same SAED pattern. Based on these indexed diffraction patterns, the crystallographic relationship between M_7_C_3_ and M_6_C could be determined as follows:

$${\left[\bar{6}515\right]}_{M_{7}C_{3}}//{\left[011\right]}_{M_{6}C}.$$

**Figure 7:**
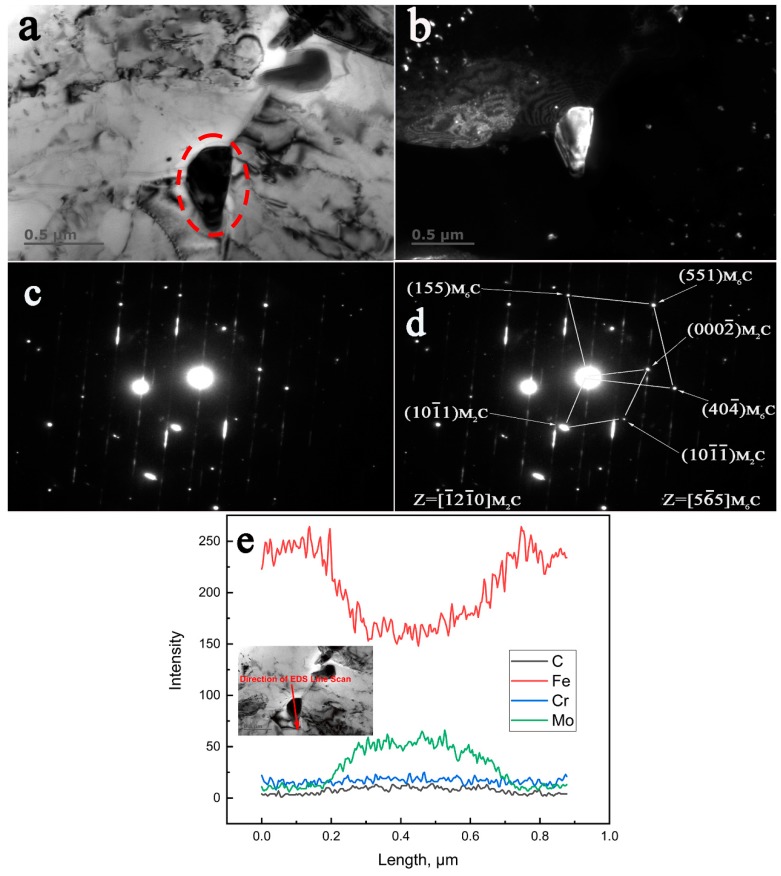
TEM characterization of the in-situ transformation of M_2_C to M_6_C in the low alloy Cr–Mo–V steel aged at 650 °C for 30,000 h. (**a**) BF image, (**b**) DF image, (**c**) the original SAED pattern, (**d**) the corresponding indexed SAED pattern, (**e**) the EDS line scan.

Similarly, a characterization of the in-situ transformed M_2_C and M_6_C in the 650 °C for 30,000 h aged specimen is shown in Figure 7. The spot used in Figure 7b was ${\left(40\bar{4}\right)}_{M_{6}C}$. The crystallographic relationship identified from the indexed SAED pattern from Figure 7d were determined as follows:

$${\left[\bar{1}2\bar{1}0\right]}_{M_{2}C}//{\left[5\bar{6}5\right]}_{M_{6}C}.$$

The corresponding original SAED pattern is shown in Figure 6c and Figure 7c, respectively. The distribution of Fe, Cr, Mo, and C in the carbide particles that precipitated when the steel was aged at 650 °C for 30,000 h can be easily seen in Figure 6e and Figure 7e.

According to the TEM and SAED analysis results, the crystallographic relationships of in-situ transformed complex precipitates in the investigated steel aged at 650 °C are summarized in Table 5. Crystallographic relationships between the in-situ transformed precipitates in the investigated low alloy Cr–Mo–V steel aged at 650 °C for the different periods. It should be pointed out that these crystallographic relationships were identified in the present study but this does not mean that all in-situ transformed carbides will transform in the same way, as there might be some other crystallographic relationships between the in-situ transformed precipitates due to the complicated and changeable environment during carbides transformation.

Interestingly, the crystallographic relationships of the in-situ transforming precipitates between the specimen aged for 13,000 h and the specimen aged for 30,000 h were different. Coincident crystallographic planes of the in-situ transforming precipitates were not observed when the specimen was aged for 30,000 h. Overlapped crystal planes, however, were observed in the specimen aged for 13,000 h.

The evidence of the in-situ transformation from M_23_C_6_ to M_6_C was not observed in this work. According to the experimental and thermodynamically calculated results of a similar low alloy Cr–Mo–V steel by Vyrostkova et al. [22,40], the M_23_C_6_ carbides prefer precipitating in larger quantity at the temperature below 600 °C. This should be the reason that the M_23_C_6_ carbides were not observed in this work.

## 4. Discussion

### 4.1. The Identification of Complex Carbide Precipitates in Long-Term Aged Cr–Mo–V Steel

In this study, three types of precipitates, namely MC, M_7_C_3_, and M_6_C, were identified in the 650 °C 13,000 h and 30,000 h aged specimens by means of X-ray diffraction. The calculation of the mass fractions of the respective precipitates by Reitveld full-pattern fitting algorithm method and TOPAS indicated the reduction of M_7_C_3_ and M_2_C precipitates with prolonged aging time at 650 °C, while the mass fractions of MC and M_6_C precipitates increased. It indicated the transition of the M_7_C_3_ and M_2_C precipitates into MC or M_6_C during aging.

The SAED investigation gave additional information about the complex precipitates, which were identified as M_7_C_3_ + M_6_C and M_2_C + M_6_C in the 13,000 h and 30,000 h aged specimens, respectively. It was confirmed by both analysis methods that the types of precipitates in the 650 °C long-term aged Cr–Mo–V steel consisted of MC, M_7_C_3_, M_6_C, and M_2_C. The present work detected the M_2_C, (M_2_C + M_6_C) for the first time, which has not been reported in previous research on similar materials [22]. Since M_2_C is a minority phase in this investigation, it could not be observed by X-ray diffraction due to the relatively low sensitivity of this technique [2]. Moreover, the size of the MC precipitate was too small to obtain its electron diffraction pattern under SAED investigation. The application of multiple inspection techniques is necessary for identifying the complex carbide precipitates in low alloy Cr–Mo–V steel.

The M_6_C precipitates were clearly detected by both X-ray diffraction and TEM methods in the long-term aged specimens in this work. When the aging treatment was not approaching the equilibrium state, such as aging between 450 °C and 650 °C from several to one thousand hours [19,23,41], the equilibrium phase of M_6_C could not be observed. The experimental evidences in the present investigation indicate that the M_7_C_3_ is the predominant carbide under the applied aging condition. This result is consistent with the theory that a relatively high Cr and Mn content in steel can accelerate the precipitation of M_7_C_3_ carbides [23,33].

### 4.2. The Sequence of Carbide Precipitation

As a very important topic of the precipitate phenomenon, the sequence of carbide precipitation has been intensively investigated. Baker and Nutting carried out pioneering work and studied the precipitation sequences during tempering of 2.25Cr–1Mo steel [31]; they clarified the precipitation sequence as:

ε-carbide → cementite → (cementite + M_2_C) → M_23_C_6_ → M_6_C;

ε-carbide → cementite → (cementite + M_2_C) → Cr_7_C_3_ → M_6_C.

Andrews, Hughes, and Dyson [42] studied the precipitation sequence at 700 °C in Cr–Mo–V rotor steels, which were summarized as follows:

Matrix → M_3_C → M_6_C;

Matrix → M_2_C → (M_2_C + M_6_C) →M_6_C.

The precipitate sequence can be influenced by many factors, such as chemical composition [17,20,22,23], original microstructure [10,16,18], aging temperature [12,13,23,31], and stress state of the steels [7]. Most research work focuses on relatively short-term aging without achieving the equilibrium state [15,22], which do not completely reveal the precipitate sequence at a given aging temperature. We applied the aging temperature of 650 °C and the aging time up to 30,000 h and approached the equilibrium state. We found the precipitation sequence for the aged Cr–Mo–V low alloy steel as follows:

MC + M_7_C_3_ + M_2_C → MC + M_2_C + M_7_C_3_ + M_6_C.

Baker and Nutting, in 1959, speculated that M_6_C may form at the expense of Mo_2_C, M_23_C_6_, or Cr_7_C_3_ [31]. To date, information about these transformations is mainly based on indirect observation, such as the similarity in the morphology and/or the chemical compositions of precipitates and sometimes the precipitation sites of these carbides [43]. This study, for the first time, provides direct evidence for the in-situ transformation of M_2_C →M_6_C and M_7_C_3_ →M_6_C (Figure 4, Figure 5, Figure 6 and Figure 7). These observations strongly support the inferences of Baker and Nutting.

According to the SAED results of the M_2_C + M_6_C complex precipitates, the M_6_C directly nucleated on the existing M_2_C and grew at the expense of M_2_C. The amount of M_2_C precipitate was limited and could not be detected by X-ray diffraction. It could be inferred that the M_2_C becomes exhausted at the equilibrium state. Based on these findings, the sequence of the carbide precipitation can be inferred as follows:

MC + M_7_C_3_ + M_2_C → MC + M_2_C + M_7_C_3_ + M_6_C → MC + M_7_C_3_ + M_6_C.

A very clear clue in the precipitation sequence of these types of carbides is that with prolonged aging the atomic percentage of carbon in the precipitates decreases. This phenomenon indicates that the dissolved carbon element in the α-Fe plays an important role in forming various carbides. At the first stage of precipitation, the dissolved carbon and the dissolved Cr and V are abundant, and the thermodynamic driving force for the evolution of MC and M_7_C_3_ is strong. With continued aging, the dissolved carbon, V, and Cr content decrease, and the dissolved Mo element becomes relatively high; then the thermodynamic driving force for the evolution of M_6_C is stronger, and M_6_C, with a low atomic percentage of carbon, turns out to be an equilibrium phase in the investigated steel. The discussion above clearly demonstrates that the localized V, Mo, Cr, and especially the carbon content in the matrix exert a decisive role in the evolution of different types of carbides. The types of equilibrium precipitates determined in the present investigation are in good agreement with the equilibrium carbides that were thermodynamically calculated by Vyrostkova and Kroupa et al. [22,33,40].

### 4.3. The Nucleation of the M_6_C Carbides

It is well known that M_6_C is a Mo rich carbide and has a face-centered cubic structure. Baker and Nutting postulated in their work that M_6_C nucleated at the interface between V_4_C_3_ and ferrite matrix [14], while Kuo and Jia found that the nucleation of M_6_C frequently occurred on M_23_C_6_ when they studied the precipitation of a low alloy Cr–Mo–V steel [30]. From Figure 6e, it can be confirmed by the EDS line scan that the up part of the carbide in the white color in the DF image is M_6_C. As shown in Figure 6b, the two white points that were represented by ‘A’ and ‘B’ can be easily recognized as M_6_C in the DF image (in this image, the M_6_C is bright and M_7_C_3_ is dark). They look like freshly nucleated carbides from their small sizes. Accordingly, this indicates that the M_6_C can nucleate at the interface of the existing M_7_C_3_ and the ferrite matrix in the aged low alloy Cr–Mo–V steel. Therefore, it can be concluded that the nucleation mechanism of M_6_C will change with the change of chemical compositions and aging conditions.

### 4.4. The Planar Mismatch between Different Phases in the Complex Precipitates

Based on the crystallographic relationships of the transformed complex carbides obtained so far in the specimen aged at 650 °C for 13,000 h, together with the inter-planar spacing of the precipitates, the lattice misfits between the phases (M_2_C and M_6_C, M_7_C_3_ and M_6_C) were calculated. The results are summarized in Table 6. It can be seen that the lattice misfit between M_2_C and M_6_C is –1.44% and that between M_7_C_3_ and M_6_C was 0.83%. These small lattice misfits indicate that the interface between the complex precipitates was coherent even when the specimen was aged for 13,000 h at 650 °C. For the specimen aged 30,000 h, however, no coherent interface between the different phases of the complex precipitates was observed. The results proposed the complex precipitates lost coherency during the aging period from 13,000 h to 30,000 h at 650 °C. The exerted strain would be released by the loss of the coherency between different phases. Miyata and Sawaragi demonstrated in their work that if the precipitates lost coherency, the positive effect of the precipitates on improving creep properties would be reduced [34]. From this point of view, it can be inferred that the creep property and toughness of the specimen aged for 30,000 h in this work will be worse than the one aged for 13,000 h.

## 5. Conclusions

The current work investigated the complex precipitates in a low alloy Cr–Mo–V steel under extreme long-term aging periods at 650 °C by combined X-ray diffraction and TEM characterization. The precipitation sequences and their crystallographic orientation relationships were established and compared to previous research findings. The main results can be summarized as follows:

1. A near-equilibrium state of the low alloy Cr–Mo–V steel was achieved by aging at 650 °C for up to 30,000 h. The transformation of M_2_C to M_6_C and M_7_C_3_ to M_6_C were observed and confirmed by means of SAED and EDS line scan. Four types of precipitates, including MC, M_7_C_3_, M_6_C, and M_2_C, were found in the specimens in the near-equilibrium state.
2. The crystallographic orientation relationships between the in-situ transformed phases of the specimen aged for 13,000 h at 650 °C were established as follows:
  $${\left(41\bar{5}\bar{1}\right)}_{M_{7}C_{3}}//{\left(\bar{4}2\bar{2}\right)}_{M_{6}C},{\left[\bar{1}2\bar{1}3\right]}_{M_{7}C_{3}}//{\left[011\right]}_{M_{6}C}$$
  $${\left(02\bar{2}1\right)}_{M_{2}C}//{\left(266\right)}_{M_{6}C},{\left[11\bar{2}\bar{6}\right]}_{M_{2}C}//{\left[31\bar{2}\right]}_{M_{6}C}.$$
3. The crystallographic orientation relationships between the in-situ transformed phases of the specimen aged for 30,000 h at 650 °C were identified as:
  $${\left[\bar{6}515\right]}_{M_{7}C_{3}}//{\left[011\right]}_{M_{6}C}\;\mathrm{and}\;{\left[\bar{1}2\bar{1}0\right]}_{M_{2}C}//{\left[5\bar{6}5\right]}_{M_{6}C}.$$
4. The carbide precipitate sequence in the investigated Cr–Mo–V steel aged at 650 °C for 30,000 h was found to be: MC + M_7_C_3_ + M_2_C → MC + M_2_C + M_7_C_3_ + M_6_C → MC + M_7_C_3_ + M_6_C, which is consistent with the equilibrium calculations.
5. The direct evidence from TEM characterization revealed that the M_6_C nucleated at the interfaces of the existing M_7_C_3_ carbides.
6. The coherent interfaces between the in-situ transformed complex carbide precipitates became incoherent at prolonged aging time of 30,000 h.

## Figures and Tables

**Table 1:** Orientation relationships between carbides and α-Fe matrix.

Carbide	Orientation Relationships with Matrix	Reference
M_3_C	${\left(100\right)}_{M_{3}C}//{\left(3\bar{1}1\right)}_{\alpha -Fe},{\left(101\right)}_{M_{3}C}//{\left(131\right)}_{\alpha -Fe},{\left(001\right)}_{M_{3}C}//{\left(\bar{2}\bar{1}5\right)}_{\alpha -Fe}$	[25]
MC (M_4_C_3_)	${\{100\}}_{M_{4}C_{3}}//{\{100\}}_{\alpha -Fe},{\langle 100\rangle }_{M_{4}C_{3}}//{\langle 100\rangle }_{\alpha -Fe}$	[26]
M_2_C	${\left(0001\right)}_{M_{2}C}//{\left(021\right)}_{\alpha -Fe},{\left(11\bar{2}0\right)}_{M_{2}C}//{\left(100\right)}_{\alpha -Fe},{\left(\bar{1}100\right)}_{M_{2}C}//{\left(01\bar{2}\right)}_{\alpha -Fe}$ ${\left(0001\right)}_{M_{2}C}//{\left(011\right)}_{\alpha -Fe},{\left(01\bar{1}1\right)}_{M_{2}C}//{\left(110\right)}_{\alpha -Fe},{\left(2\bar{1}\bar{1}0\right)}_{M_{2}C}//{\left(1\bar{1}1\right)}_{\alpha -Fe}$	[27] [28]
M_7_C_3_	${\left(0001\right)}_{M_{7}C_{3}}//{\left(011\right)}_{\alpha -Fe},{\left(10\bar{1}0\right)}_{M_{7}C_{3}}//{\left(11\bar{1}\right)}_{\alpha -Fe},{\left(01\bar{1}0\right)}_{M_{7}C_{3}}//{\left(\bar{2}3\bar{3}\right)}_{\alpha -Fe}$ ${\left(1\bar{1}00\right)}_{M_{7}C_{3}}//{\left(100\right)}_{\alpha -Fe},{\left(0001\right)}_{M_{7}C_{3}}//{\left(21\bar{1}\right)}_{\alpha -Fe},{\left(10\bar{1}0\right)}_{M_{7}C_{3}}//{\left(102\right)}_{\alpha -Fe}$ ${\left(01\bar{1}0\right)}_{M_{7}C_{3}}//{\left(2\bar{3}1\right)}_{\alpha -Fe},{\left(1\bar{1}00\right)}_{M_{7}C_{3}}//{\left(011\right)}_{\alpha -Fe}$	[29]
M_6_C	${\left(100\right)}_{M_{6}C}//{\left(100\right)}_{\alpha -Fe},{\left(011\right)}_{M_{6}C}//{\left(011\right)}_{\alpha -Fe},{\left(0\bar{1}1\right)}_{M_{6}C}//{\left(0\bar{1}1\right)}_{\alpha -Fe}$	[30]
M_23_C_6_	${\left(011\right)}_{M_{23}C_{6}}//{\left(011\right)}_{\alpha -Fe},{\left(\bar{1}1\bar{1}\right)}_{M_{23}C_{6}}//{\left(110\right)}_{\alpha -Fe},{\left(\bar{2}\bar{1}1\right)}_{M_{23}C_{6}}//{\left(\bar{1}10\right)}_{\alpha -Fe}$	[30]

**Table 2:** Crystallographic information of typical carbides in low alloy Cr–Mo–V steel.

Carbide	Lattice Parameters (nm)	Crystal System	Space Group	Reference
M_3_C	a = 0.4525 b = 0.5087 c = 0.6743	orthorhombic	Pnma	[35]
M_6_C	a = 1.1082	cubic	Fd $\bar{3}$ mm	[35]
M_23_C_6_	a = 1.0621	cubic	Fm $\bar{3}$ m	[35]
MC	a = 0.8333	cubic	P4_3_32	[36]
M_2_C	a = 0.3002 c = 0.4724	hexagonal	P6_3_/mmc	[37]
M_7_C_3_	a = 0.4526 b = 0.7010 c = 1.2142	orthorhombic	Pnma	[38]

**Table 3:** Chemical composition of the investigated low alloy Cr–Mo–V steel (in wt%).

C	Mn	P	S	Cr	Mo	Nb	V	Al	N
0.14	0.55	0.008	0.003	2.5	1.0	0.03	0.3	0.020	0.005

**Table 4:** The types of the precipitates from the 650 °C aged specimens as identified by X-ray diffraction measurements.

Aging Temperature (°C)	Aging Time (h)	Types of Carbides
650	0	M_7_C_3_ + MC + M_2_C
650	13,000	M_7_C_3_ + MC + M_6_C
650	30,000	M_7_C_3_ + MC + M_6_C

**Table 5:** Crystallographic relationships between the in-situ transformed precipitates in the investigated low alloy Cr–Mo–V steel aged at 650 °C for the different periods.

Aging Time (h)	In-Situ Transformation	Crystallographic Relationships between the Precipitates
13,000	M_7_C_3_ → M_6_C	$\begin{array}{l} {\left(41\bar{5}\bar{1}\right)}_{M_{7}C_{3}}//{\left(\bar{4}2\bar{2}\right)}_{M_{6}C}\\ {\left[\bar{1}2\bar{1}3\right]}_{M_{7}C_{3}}//{\left[011\right]}_{M_{6}C} \end{array}$
	M_2_C → M_6_C	$\begin{array}{l} {\left(02\bar{2}1\right)}_{M_{2}C}//{\left(266\right)}_{M_{6}C}\\ {\left[11\bar{2}\bar{6}\right]}_{M_{2}C}//{\left[31\bar{2}\right]}_{M_{6}C} \end{array}$
30,000	M_7_C_3_ → M_6_C	${\left[\bar{6}515\right]}_{M_{7}C_{3}}//{\left[011\right]}_{M_{6}C}$
	M_2_C → M_6_C	${\left[\bar{1}2\bar{1}0\right]}_{M_{2}C}//{\left[5\bar{6}5\right]}_{M_{6}C}$

**Table 6:** Lattice misfits between different phases of the transformed complex precipitates of the specimen aged at 650 °C for 13,000 h.

Face	(hkil)_carbide_	Lattice Spacing, d_1_, nm	(hkl)_carbide_	Lattice Spacing, d_2_, nm	Lattice Misfits, (d_1_ – d_2_)/d_1_
${\left(41\bar{5}\bar{1}\right)}_{M_{7}C_{3}}//{\left(\bar{4}2\bar{2}\right)}_{M_{6}C}$	${\left(41\bar{5}\bar{1}\right)}_{M_{7}C_{3}}$	0.2281	${\left(\bar{4}2\bar{2}\right)}_{M_{6}C}$	0.2262	0.83%
${\left(02\bar{2}1\right)}_{M_{2}C}//{\left(266\right)}_{M_{6}C}$	${\left(02\bar{2}1\right)}_{M_{2}C}$	2 × 0.1253	${\left(266\right)}_{M_{6}C}$	0.2542	−1.44%

## References

[1] Bhadeshia H.K.D.H. (2001). Design of Ferritic Creep-resistant Steels. ISIJ Int..

[2] Mitchell D.R.G., Ball C.J. (2001). A quantitative X-ray diffraction and analytical electron microscopy study of service-exposed 2.25Cr-1Mo steels. Mater. Charact..

[3] Wang Y., Cheng G., Qin M., Li Q., Zhang Z., Chen K., Li Y., Hu H., Wu W., Zhang J. (2017). Effect of high temperature deformation on the microstructure, mechanical properties and hydrogen embrittlement of 2.25Cr–1Mo-0.25 V steel. Int. J. Hydrogen Energy.

[4] Pereira P.A.S., Franco C.S.G., Guerra Filho J.L.M., Dos Santos D.S. (2015). Hydrogen effects on the microstructure of a 2.25Cr-1Mo-0.25 V steel welded joint. Int. J. Hydrogen Energy.

[5] Chen J.B., Liu H.B., Pan Z.Y., Shi K., Zhang H.Q., Li J.F. (2015). Carbide evolution and service life of simulated post weld heat treated 2.25Cr-1Mo steel. Mater. Sci. Eng. A.

[6] Taneike M., Abe F., Sawada K. (2003). Creep-strengthening of steel at high temperatures using nano-sized carbonitride dispersions. Nature.

[7] Yu X., Babu S.S., Terasaki H., Komizo Y., Yamamoto Y., Santella M.L. (2013). Correlation of precipitate stability to increased creep resistance of Cr-Mo steel welds. Acta Mater..

[8] Nguyen T.D., Sawada K., Kushima H., Tabuchi M., Kimura K. (2014). Change of precipitate free zone during long-term creep in 2.25Cr-1Mo steel. Mater. Sci. Eng. A.

[9] Dépinoy S., Toffolon- Masclet C., Urvoy S., Roubaud J., Marini B., Roch F., Kozeschnick E., Gourgues A.F. (2017). Carbide precipitation in 2.25 Cr-1 Mo bainitic steel: Effect of heating and isothermal tempering conditions. Metall. Mater. Trans. A.

[10] Zhang Y., Luo P., Yan H., Zhang H., Li J. (2018). The Effect of Bainite Type on the Evolution of Carbide Constituent During an Accelerated Aging in Cr-Mo-V Steel. J. Mater. Eng. Perform.

[11] Wang X., Li Y., Li H., Lin S., Ren Y. (2018). Effect of long-term aging on the microstructure and mechanical properties of T23 steel weld metal without post-weld heat treatment. J. Mater. Process Tech..

[12] Jiang Z., Wang P., Li D., Li Y. (2019). Influence of the decomposition behavior of retained austenite during tempering on the mechanical properties of 2.25Cr-1Mo-0.25V steel. Mater. Sci. Eng. A.

[13] Zieliński A., Golański G., Sroka M. (2017). Influence of long-term ageing on the microstructure and mechanical properties of T24 steel. Mater. Sci. Eng. A.

[14] Baker R.G., Nutting J. (1959). The tempering of a Cr-Mo-V-W and a Mo-V steel. Iron and Steel Inst. Spec. Rep..

[15] Janovec J., Vyrostkova A. (1992). Effect of tempering on development of carbide particles in 2.7Cr-0.6Mo-0.3V steel. J. Mater. Sci..

[16] Ishiguro T., Ohnishi K., Watanabe J. (1986). Effects of chromium and vanadium on the hydrogen attack susceptibility of boron added Cr-Mo steels. Tetsu-to-Hagane.

[17] Vodarek V., Strang A. (1997). Effect of nickel on the precipitation processes in 12CrMoV steels during creep at 550 °C. Scripta Mater..

[18] Jiang Z.H., Wang P., Li D.Z., Li Y.Y. (2017). The evolution of microstructure and mechanical properties of 2.25Cr-1Mo-0.25V steel with different initial microstructures during tempering. Mater. Sci. Eng. A.

[19] Zhang Y., Miao L., Wang X., Zhang H., Li J. (2009). Evolution behavior of carbides in 2.25Cr-1Mo-0.25V steel. Mater. Trans..

[20] Wen T., Hu X.F., Song Y.Y., Yan D.S., Rong L.J. (2013). Carbides and mechanical properties in a Fe-Cr-Ni-Mo high strength steel with different V contents. Mater. Sci. Eng. A.

[21] Seung P.H., Seong I.K., Tae Y.A., Soon T.H., Young W.K. (2016). Effects of extended heat treatment on carbide evolution in Cr-Mo steels. Mater. Charact..

[22] Vyrostkova A., Kroupa A., Janovec J., Svoboda M. (1998). Carbide reactions and phase equilibria in low alloy Cr-Mo-V steels tempered at 773-993 K. Part I: Experimental measurements. Acta Mater..

[23] Yu J. (1989). Carbide stability diagrams in 2.25Cr-1Mo Steels. Metall. Trans. A.

[24] Senior B.A. (1988). A critical review of precipitation behaviour in 1Cr-Mo-V rotor steels. Mater. Sci. Eng. A.

[25] Pitsch W. (1962). The orientation relationship between cementite and ferrite in pearlite. Acta Metall..

[26] Heikkinen V.K., Hakkarainen T.J. (1973). Precipitation associated with the climb of a <100> dislocations in a low-carbon iron-vanadium alloy. Philos. Mag..

[27] Rong W., Dunlop G.L. (1984). The crystallography of secondary carbide precipitation in high speed steel. Acta Metall..

[28] Lee T., Oh C., Ryu S., Kim J. (2011). Crystallography and morphology of carbides in a low-cycle fatigued 1Cr-1Mo-0.25V steel. Metall. Mater. Trans. A.

[29] Dyson D.J., Andrews K.W. (1969). Carbide M_7_C_3_ and its formation in alloy steels. J. Iron Steel Inst..

[30] Kuo K.H., Jia C.L. (1985). Crystallography of M_23_C_6_ and M_6_C precipitated in a low alloy steel. Acta Mater..

[31] Baker R.G., Nutting J. (1959). The tempering of 2.25Cr-1Mo steel after quenching and normalizing. J. Iron Steel Inst..

[32] Woodhead J.H., Quarrell A.G. (1965). Role of carbides in low-alloy creep resisting steels. J. Iron Steel Inst..

[33] Kroupa, Havránková J., Svoboda M., Coufalová M., Vřešt’Ál J. (2001). Phase diagram in the iron-rich corner of the Fe-Cr-Mo-V-C system below 1000 K. J. Phase Equ..

[34] Miyata K., Sawaragi Y. (2001). Effect of Mo and W on the phase stability of precipitates in low Cr heat resistant steels. ISIJ Int..

[35] Bhadeshia H.K.D.H., Christian J.W. (1990). Bainite in steels. Metall. Trans. A.

[36] Rempel A.A. (1996). Atomic and vacancy ordering in nonstoichiometric carbides. Physics-Uspekhi.

[37] Andrews K.W., Dyson D.J., Keown S.R. (1967). Interpretation of Electron Diffraction Pattern.

[38] Morniroli J.P., Bauer-Grosse E., Gantois M. (1983). Crystalline defect in M_7_C_3_ carbide. Phil. Mag. A.

[39] Zhang Y.T., Han H.B., Miao L.D., Zhang H.Q., Li J.F. (2009). Quantitative carbide analysis using the Rietveld method for 2.25Cr-1Mo-0.25V steel. Mater. Charact..

[40] Kroupa A., Vyrostkova A., Svoboda M., Janovec J. (1998). Carbide reactions and phase equilibria in low alloy Cr-Mo-V steels tempered at 773-993 K. Part II: Theoretical calculations. Acta Mater..

[41] Fu R.D., Wang T.S., Zhou W.H. (2007). Characterization of precipitates in a 2.25Cr-1Mo-0.25V steel for large-scale cast-forged products. Mater. Charact..

[42] Andrews K.W., Hughes H., Dyson D.J. (1972). Constitution diagrams for Cr-Mo-V steels. J. Iron Steel Inst..

[43] Inoue A., Masumoto T. (1980). Carbide reactions (M3C→M7C3→M23C6→M6C) during tempering of rapidly solidified high carbon Cr-W and Cr-Mo steels. Metall. Trans. A.

